# Regulated, reliable, and reputable: Protect patients with uniform standards for stem cell treatments

**DOI:** 10.1002/sctm.19-0377

**Published:** 2020-02-10

**Authors:** Geoffrey P. Lomax, Art Torres, Maria T. Millan

**Affiliations:** ^1^ California Institute for Regenerative Medicine Oakland California

**Keywords:** clinical trials, gene therapy, stem cells, stem cell transplantation, ethics

## Abstract

The promise of cell and gene therapies is being realized as new products emerge to treat diseases once considered intractable. These treatments are emerging amidst reports of patients being injured by unproven “stem cell” interventions. At this juncture, it is vital to be supporting the continued development of promising regenerative medicine products while protecting patients from the risks posed by unproven interventions. Various stakeholders, including governments, patient groups, medical societies, and the media, are committed to this outcome. In this perspective, we draw on our experience gained from partnerships in developing regenerative medicine products to identify technical, organizational, and ethical benchmarks for the responsible delivery of regenerative medicine treatments. These benchmarks may serve as the basis for policy interventions intended to drive the responsible delivery of stem cell and regenerative medicine products. Our particular focus is on a California‐based policy, but the suggested benchmarks are broadly applicable to national and international jurisdictions.

**Significance statement**

This perspective is aimed at building consensus for the stem cell field to ensure proper conduct of clinical research and the regulation of the practice of medicine involving cell‐based treatments.

## 1 INTRODUCTION

We are at a turning point in the field of stem cell science and regenerative medicine, with rigorous science giving rise to new treatments for patients in need. The first wave of cell and gene medicine programs has obtained the US Food and Drug Administration (FDA), European Commission, or other regulatory body marketing approval. These products include gene therapies for inherited blindness, beta‐thalassemia, and a fatal form of infant paralysis in addition to gene‐engineered immune cells that target resistant blood cancers. In mid‐2019, there were 366 gene therapies and 410 gene‐engineered cell therapies in clinical trials, and the FDA has approved 17 products.^1^, ^2^

Concomitant with the advance of scientifically rigorous clinical research programs has been the proliferation of clinics offering unproven interventions internationally.^3^ For the purpose of this perspective, “unproven” interventions refer to cell‐ and tissue‐based products marketed directly to patients that would (i) be considered biological products by the FDA and regulated as such and/or (ii) have not been evaluated to characterize product composition, safety, or efficacy for the intended indication.^4^

Case reports have documented such interventions have resulted in injuries and deaths in patients, and they are often administered by clinicians practicing outside their medical training.^5^, ^6^, ^7^, ^8^ Patients report coercive direct‐to‐consumer marketing of interventions, costing thousands of dollars, for which clinical efficacy has not been established.^9^, ^10^, ^11^ Collectively, these practices increase the potential for patient exploitation and harm through the imposition of medical and financial risks.^12^

Recognizing these potential harms, actions have been taken by different stakeholders to protect patients from the medical and financial risks posed by unproven interventions.

- Traditional regulatory bodies such as the FDA, European Medicines Agency (EMA), and the US Federal Trade Commission (FTC) have taken enforcement actions.^12^, ^13^, ^14^
- The International Society of Stem Cell Research educates patients to identity “red flags” attributed to unproven interventions.^15^
- In the US states, medical boards have considered options for ensuring patient safety and attorneys general have investigated and/or sought injunctions against clinics marketing unproven interventions. 16, 17
- The US states have considered and passed laws requiring clinics to disclose to patients if a practitioner is offering cell products not approved or authorized by the FDA.^18^, ^19^
- Patient and patient organizations have become increasingly engaged in advocacy to access the best treatment options for individual with unmet medical needs.^20^
- Google has announced a new policy on advertising for speculative and experimental medical treatments with an emphasis on stem cell treatments. The Google policy prohibits ads selling treatments that *have no established biomedical or scientific basis* and includes treatments *that are rooted in basic scientific findings and preliminary clinical experience, but currently have insufficient formal clinical testing to justify widespread clinical use*.^21^

These steps are illustrative of the multi‐stakeholder approach needed to simultaneously advance promising new patient treatments while deterring the marketing of products with no demonstrated medicinal value.

## 2 CIRM'S ADVOCACY FRAMEWORK

The established US system of regulation governing stem cell and regenerative medicine products is intended to simultaneously spur innovation while protecting the safety and welfare of patients.^22^ The focus is on compiling evidence to document the composition and manufacturing of products, demonstrate safety and efficacy, and inform marketing and distribution.^23^ In the United States, however, states enact laws and regulations that govern the practice of medicine within their borders.^24^ Thus, some practitioners argue that certain interventions performed within states, such as the collection and subsequent injection of autologous cells and tissue, are exempt from FDA regulation.^25^ Thus, it is important to augment existing federal regulations with state‐based initiatives to protect patients.

In California, efforts have been made to improve the oversight of clinics offering stem cell treatments. A proposed 2019 state law would have created a Regulatory Advisory Group to make recommendations to the Legislature for how the oversight of stem cell treatments could be improved.^26^ In this legislative context, the CIRM has identified a series of technical, organizational, and ethical benchmarks that may be utilized to support uniform policies to support the responsible delivery of stem cell treatments.^27^ The CIRM recommends California adopt a policy framework to ensure stem cell products are regulated, reliable, and reputable.

**Figure 1:**
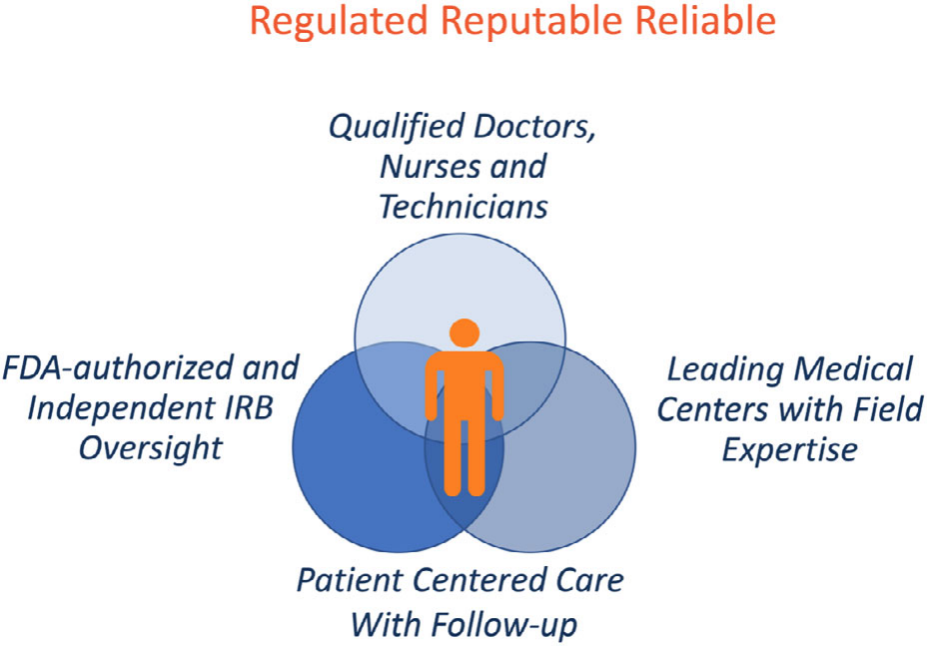
A framework for the delivery of regenerative medicine

The moniker of regulated, reliable, and reputable is intended to encapsulate the milieu of scientific, ethical, and legal considerations into a framework that can inform policies to support the responsible delivery of stem cell and regenerative medicine products (Figure 1). Although our focus is on state‐based policy, commentators have consistently suggested a more uniform international approach to advancing promising treatments while deterring unproven intervetions.^28^

This framework suggests a set of benchmarks the multitude of stakeholders may apply to inform policy interventions at the product, practitioner, and organizational level. Under this framework, stem cell and regenerative medicine products should:

1. adhere to regulatory standards (product level),
2. be administered by reliable and qualified teams of practitioners (practitioner level), and
3. be delivered at reputable medical centers (organizational level).

State‐based policies governing stem cell treatments at the practitioner and organizational level are comparatively underdeveloped in relation to the FDA regulation of products. Table 1 illustrates a variety of regulatory policy considerations and distinguishes between existing or established standards and suggests potential options for expansion. The intent is to suggest that a comprehensive framework to advance a standard of care for the delivery of treatments to patients will require expansion of existing policies in a multi‐stakeholder approach. In the case of the United States, such an approach will likely require further action at the state level, and specific benchmarks are suggested to support policy deliberations. Furthermore, these same benchmarks may be applied in international jurisdictions according to their systems of medical regulation.

**Table 1:** Regulatory and policy considerations for enhancing patient safety

Considerations	Authority	Level of intervention	Benchmark (established and potential)
**Manufacturing and Processing Standards**: International standards exist for the manufacturing and processing of human cells and tissue that ensure their safety and quality. Any establishment providing human cells or tissue in the United States must be registered with the FDA	Pan‐national WHO/EMA/ISO/Pharmaceutical Inspection Convention National (US FDA) State‐based facilities licensing	Product	Applicable product certification requirements are met^a^ Establishment manufacturing or processing cell or tissue is FDA‐registered^a^ Establishment is licensed in state^a^
**Product Authorization**: Administration of the product to patients should be authorized by an independent authority	National (US FDA)	Product	Product is FDA‐approved^a^ Product being studied under an IND^a^ Product meets FDA minimal manipulation and homologous use criteria^a^
**Certified Clinicians**: Doctors providing treatment should be certified by specialty area	State medical boards	Practitioner	Board‐certified in treatment area^b^
**Specialized Training**: Nurses and technicians possess specialized training and expertise	Certification and credential programs	Practitioner	Certificate of training in cell therapy^b^
**Disclosure**: Provide essential information consistent with standards for voluntary informed consent	OHRP/Common Rule State laws for human subjects protection	Organizational	Disclosure of risks and benefits consistent with Common Rule^b^
**Notification**: Patient told of any procedure not FDA‐authorized or FDA‐approved	State laws	Organizational	Clear notification provided^a^
**Standards of Evidence**: Claims of efficacy should be supported by published peer‐reviewed evidence	State medical boards Standard of practice	Organizational	Claims supported by independent medical journal^b^
**Surveillance**: Clinic should monitor patient for safety	State medical boards Standard of practice	Organizational	Adverse outcomes are systematically documented^b^
**Registry of Outcomes**: Public database for patient or provided reported outcomes	State laws/public agency	Product, organization, or practitioner	Adverse outcomes are systematically documented^b^
**Freedom to Disclose**: Patients should not be restricted in their right to talk about a medical practice or stem cell treatment	State laws	Patient (autonomy)	No gag‐rule laws in place^b^

a *Established*: Existing standard, law, or policy.

b *Potential*: No current standard, law, or policy.

## 3 ADHERENCE TO REGULATORY STANDARDS

The regulation of drugs has historically been driven by concerns over unsafe or “adulterated” products being sold to patients.^29^ The FDA and other regulatory bodies have three primary concerns: (a) safety and effectiveness in the product's proposed use; (b) appropriateness of the proposed labeling; and (c) adequacy of manufacturing methods to assure the product's identity, strength, quality, and purity.^30^ CIRM has embedded regulatory requirements into its clinical research and infrastructure programs such as the CIRM Alpha Stem Cell Clinics Network.^31^ CIRM is supporting, directly and indirectly, over 125 clinical trials in a multitude of disease areas suggesting the practicability and feasibility of this evidence‐based approach while enabling patient access to new treatments for unmet medical needs.^32^

### 3.1 Manufacturing and processing

Stem cell treatments are often human cells, tissues, or tissue‐based products. There are internationally established standards according to Good Manufacturing Practice for pharmaceutical manufacturers.^33^ The FDA requires that specific safety procedures be followed in the collection, processing, and delivery of these products.^34^ The FDA and US states license facilities that process human blood and tissue.^35^ In addition, the FDA has established a database of registered human cell and tissue establishments.^36^ The FDA has issued warnings to clinics about the risk posed by unapproved cellular products that do not conform to these safety procedures or registration requrements.^14^ The CIRM requires clinical trials sponsors to document compliance with the FDA and California standards and certifications for manufacturing, processing, and registration.

- Stem cell or regenerative medicine products should conform to the FDA (or equivalent) and state standards for manufacturing, processing, and facility registration.

### 3.2 Product authorization

In the United States, an Investigational New Drug Application (IND) is a request for FDA authorization to administer a new drug to humans. The primary purpose of the IND submission is to ensure that patients will not face undue risk of harm, in part, by conforming to manufacturing and processing standards.^37^ Furthermore, the IND process establishes a system where clinical evidence must be gathered and subsequently evaluated by the FDA. All clinical trials conducted with CIRM funding or being conducted in CIRM's Alpha Stem Cell Clinics Network must be authorized by the FDA (IND).

- The administration of any stem cell or regenerative medicine product should be authorized or approved by the FDA.

## 4 RELIABLE PRACTITIONERS

### 4.1 Certified clinicians

Doctors, nurses, and technicians providing stem cell treatments should possess specialized training and expertise. In the United States, state medical boards certify doctors according to specialty areas, and the Federation of State Medical Boards maintains a Physician Data Center.^38^ The American Board of Medical Specialties certifies doctors in a number of specialty areas.^39^ Having the proper credentials means the doctors, nurses, and other experts involved can reliably administer treatments and follow up with the patient. Because stem cell treatments are a comparatively new area of medicine, specialty area expertise and clinical experience are important attributes for detecting indications that the patient may be having an unanticipated response to a treatment. There are specialized training and accreditation programs for doctors and the organizations providing stem cell treatments.^40^ Having a degree is not necessarily sufficient. A CIRM Alpha Clinic will only support a clinical research trial when it is directed by a doctor with certification in the specialty area for that disease.

- Administration of stem cell or regenerative medicine products should be directed by a doctor with certification in the particular specialty area for that disease or condition.

In contrast, reports suggest that some unproven interventions are administered by doctors that are not credentialed in the specialty areas.^41^ In addition, the individuals handling cell or cell‐products may not have appropriate expertise or training. For example, the FDA recently issued a warning to a cord and umbical cord blood processor for not following proper manufacturing practices.^42^

## 5 REPUTABLE CLINICAL OPERATIONS

Reputable medical providers will maintain systems and operating procedures designed to educate and evaluate patients through the treatment process. Essential elements include disclosure and consent, patient follow‐up, and adverse event reporting.

### 5.1 Patient disclosure and consent

One way the rights and welfare of patients are protected is by providing complete information about the potential risks and benefits of a clinical trial. This information is disclosed through the informed consent process. There are established standards of care for providing informed consent.^43^ Much of the information provided in the consent process is fundamental to all medical decision‐making. Patients need to know the potential risks and benefits of a treatment as well as alternatives. Organizations such as the International Society for Stem Cell Research, the National Academies of Sciences, Engineering and Medicine, and the CIRM have endorsed providing complete disclosure when stem cell‐based treatments are offered outside a formal clinical trial.^44^

- The medical standard of care for providing stem cell treatments outside a formal clinical trial should include disclosure of essential information consistent with standards for voluntary informed consent.

There may be circumstances where a procedure involves minimally manipulated cells for homologous use.^3^ In this case, the product may not be authorized or approved by the FDA. In California, if a practitioner is offering cell products not approved or authorized by the FDA, then the patient must be notified in writing of the following:

THIS NOTICE MUST BE PROVIDED TO YOU UNDER CALIFORNIA LAW. This health care practitioner performs one or more stem cell therapies that have not yet been approved by the United States Food and Drug Administration. You are encouraged to consult with your primary care physician prior to undergoing a stem cell therapy.^45^

- Patients should be notified of any stem cell procedure involving minimally manipulated cells not authorized or approved by the FDA.

### 5.2 Claims of clinical effectiveness

Clinics providing unproven interventions often exaggerate claims of medical benefit from the product. Common exaggerations include the ability to treat a wide range of diseases. For example, one clinic that was fined for false advertising by the FTC claimed a single product could treat Parkinson's disease, multiple sclerosis, cerebral palsy, macular degeneration, osteoarthritis, strokes, heart attacks, and chronic kidney disease.^46^ Patient queries will often result in “sales‐pitches” from the clinic.^12^ Clinics providing unproven interventions will often rely on patient testimonials to support claims of effectiveness.^47^ These testimonials may be found on clinic websites, blogs, social media sites, and are uploaded onto YouTube. A review of information provided by clinics offering unproven interventions suggested they are not making a serious effort to draw on or expand the body of scientific knowledge by publishing objective results.^48^

- Any claims of efficacy should be supported by clinical data published in peer‐reviewed journals.

### 5.3 Follow‐up and adverse event reporting

As part of the IND process, the FDA will require the collection and submission of clinical information to monitor patient safety. Often, specialized data safety monitoring boards evaluate this information to protect current and future patients. Based on this monitoring, the treatment protocol may be modified to enhance safety. In addition, because stem cell and gene therapy treatments can persist in the human body, the FDA and other regulatory bodies recommend plans for the long‐term monitoring of patients.^49^, ^50^ All clinical trials conducted with CIRM‐funding or being conducted in CIRM's Alpha Stem Cell Clinics Network provide patient support and post‐treatment follow‐up.

- Clinics providing stem cell or regenerative medicine products should provide ongoing support and follow‐up of patients including monitoring for safety and efficacy.

Objective measures of efficacy should exist for a particular indication and patients should be evaluated against these benchmarks. Patients should also be monitored or encouraged to report any adverse events. Bleeding, pain, infection, and inflammation have been associated with these treatments.^51^ In addition, any conditions requiring patient hospitalization should be documented. Recognizing there may be disincentives for providers to report and to enhance safety, medical boards should have mechanisms to receive and investigate reports, from patients or other medical providers, of adverse events.

- Patients and or provider reported outcome data should be compiled and available for ongoing evaluation of safety and efficacy.

### 5.4 Patient autonomy

Serious safety concerns resulting from unanticipated responses to unproven stem cell treatments have been documented.^51^ Such adverse effects are probably more common than is appreciated, because there is no reporting requirement when these interventions are administered outside clinical trial investigations.^52^ Also, there have been examples of medical providers requiring patients to sign agreements requiring them to not talk negatively about a practice.^53^ There is no formal mechanism for reporting and documenting outcomes of unproven stem cell treatments both positive and negative. A cell therapy registry for reporting outcomes could improve the current state of information and serve to better inform patients about stem cell treatments.

- Patients should not be restricted in their right to talk about a medical practice or stem cell treatments.

## 6 CONCLUSIONS

There are documented examples of unproven stem cell interventions causing harm to patients. In the majority of examples, the intervention deviates from the norms of responsible medical practice. Numerous authoritative bodies have raised concerns over the potential for medical and financial harm to result from these practices. In the United States, it is vital that the US FDA, the FTC, state medical boards, medical licensing and accreditation organizations, and state consumer protection bodies have the authority to promulgate policies to prevent unproven interventions and advance appropriate care consistent with the principles of good clinical research. In this perspective, we have outlined a series of policy interventions and benchmarks to support these objectives. Organizations that advertise or report on medical treatments may utilize these principles and benchmarks for evaluating the legitimacy of stem cell and regenerative medicine treatments. CIRM encourages all stakeholders to work collaboratively to advance policies that drive the responsible delivery of stem cell and regenerative medicine products.

## CONFLICT OF INTEREST

The authors declared no potential conflicts of interest.

## AUTHOR CONTRIBUTIONS

G.P.L.: conception and design, collection and/or assembly of data, manuscript writing; A.T.: conception and design, final approval of manuscript; M.T.M.: conception and design, manuscript writing, final approval of manuscript.

## Data Availability

Data sharing is not applicable to this article as no new data were created or analyzed in this study.
